# Effects of Processing on Chemical Composition of Extracts from Sour Cherry Fruits, a Neglected Functional Food

**DOI:** 10.3390/antiox12020445

**Published:** 2023-02-10

**Authors:** Francesco Cairone, Caterina Fraschetti, Luigi Menghini, Gokhan Zengin, Antonello Filippi, Maria Antonietta Casadei, Stefania Cesa

**Affiliations:** 1 Department of Drug Chemistry and Technology, “Sapienza” University of Rome, P.le Aldo Moro 5, 00185 Rome, Italy; 2 Department of Pharmacy, G. d’Annunzio, University of Chieti-Pescara, 66013 Chieti, Italy; 3 Department of Biology, Science Faculty, Selcuk University, Konya 42130, Turkey

**Keywords:** sour cherry, “visciola di Sezze”, HPLC-DAD analysis, DI-ESI-MS analysis, CIEL*a*b* analysis, DPPH analysis, *Prunus cerasus* L.

## Abstract

Sour cherries fruits (*Prunus cerasus* L., syn *P. cerasus* var. *austera*) are locally known as “visciola di Sezze”, due to the name of the city where they are traditionally cultivated in Lazio Region, Italy. Fruit samples from three harvesting years (June 2019, 2020 and 2021), were submitted to a protocol of analyses to detect the bioactive content based on year of harvest, freezing, homogenization and thermic treatments. Polyphenolic components, particularly anthocyanin compounds, were extracted, purified and analyzed by HPLC-DAD and DI-ESI-MS. An anthocyanin content between 0.24 and 21 mg/g fresh weight and a flavonols content between 0.04 and 0.2 mg/g fresh weight were found, depending on the harvest year and the applied procedures. Anthocyanins, besides being the principal components, were mainly represented by cyanidin-3-glucosyl-rutinoside (about 80%), a not particularly widespread molecule, mostly accounting for polyphenolic content. Color analysis and anti-radical activity of the different obtained extracts were performed with the aim to correlate organoleptic characters and health potential to the detected anthocyanins and flavanols content. Results show that immediate post-harvest freezing is the best way to preserve the bioactive content, the correlated color expression and anti-radical activity.

## 1. Introduction

Sour cherry (*Prunus cerasus* L.) is a tree belonging to the Rosaceae family that produce small reddish fruit that are similar in shape to the sweet cherry. Not widely used as fresh fruits, the production of sour cherry is consistent and mainly oriented to processed products, such as dry or frozen fruits, juice, liquors and marmalade with characteristic organoleptic properties. The fruits are drupes of spheroid shape, with a thin epicarp and fleshy pulp that evolve with ripening into an intense pale-to-dark reddish color, depending on the selected variety [1,2].

The taxonomy of *Prunus cerasus* L. includes orchard varieties known for food use such as black cherry (characterized by pale red skin and yellow juicy flesh, local name *amarena*), sour black cherry (with reddish-black epicarp and pulp, local name *marasca*) and sour cherry (with deep red peel as well as the soft and juicy flesh; it was previously identified as *P. cerasus* L. ‘austera’, also known as morello cherry or *visciola*), which is characterized by acid taste and the smallest fruits, especially if compared to the more appreciated sweet cherry. Sour cherry fruits, due to the characteristic taste result of low palatability, are mainly processed as dry/frozen fruits, liquors or marmalade. Intriguingly, the limited appeal for fresh consumption can be related to the relevant amounts of secondary metabolites, while the phytocomplex results have only marginally been investigated [1]. 

In fact, if a highly limited seasonal availability largely influences the processing of other different berries (blueberries, strawberries and sweet cherries) in order to obtain preserves, jams and juices [3,4], in the case of sour cherries, processing is also adopted in response to their poor palatability. A scarce pleasantness is in fact linked to the strongly acidic character, and different transformed products are prepared with the aim to meet the consumers’ needs.

Relatively few papers deal with the study of this food matrix. Turkish, Polish and Hungarian cultivars, which represent the most cultivated worldwide, were studied for their polyphenolic and anthocyanin content, color expression and thermal induced modification during processing [5,6,7,8,9].

Despite the less appreciated organoleptic properties, sour cherry represents a concentrate of nutritive (simple sugars and small quantities of proteins) and non-nutritive molecules (vitamins A, B_1_, B_2_, B_3_ and C; minerals K, Fe, Ca, P), among which polyphenols, anthocyanins and flavonols which highly correlate with antioxidant, anti-radical and disease preventing potential [6,9]. Besides the polyphenolic content, sour cherries present a very peculiar anthocyanin component deserving of exploration. In fact, if the most represented anthocyanidin is cyanidin, one of the most diffuse pigments in vegetables, this is here represented in different glycosylated forms, such as 3-sophoroside, 3-rutinoside (CR) and especially 3-glucosyl-rutinoside (CGR), not yet represented in other matrices [10]. Identified in Montmorency sour cherries in 1970, by Fischer and Von Elbe [11], this last anthocyanin represents the object of a very limited number of papers. The few available studies on this topic show, on the whole, a clear prevalent presence of CGR with respect to CR in tart cherries; only small quantities of this molecule are reported in raspberries and sweet cherries in which cyanidin-3-rutinoside is mainly represented [12].

In stark contrast to its scarce occurrence in nature, CGR can represent in sour cherry more than 80–90% of the total anthocyanin content, the anthocyanin compounds being the main components of the flavonoid and of the polyphenols fraction of this food matrix [13]. 

Some limited papers report the occurrence of CGR in other less studied and diffused sources, such as Surinam cherries, red currant and cloudberry [14,15,16]. Few studies have been performed specifically on the CGR and other cyanidin-glucosides’ bioactivity. Seeram et al. [12] reported that the antioxidant activity of cyanidin glycosides increases with a decreasing number of sugar units. Martin et al. in a relatively recent paper [17], report, quite unexpectedly, the activity of CGR as AMPK (AMP activated protein kinase) inhibitor with an IC_50_ of 3.2 μM, roughly comparable to that of ellagic acid (0.67 μM), a molecule already known for its modulating activity on protein kinases. This recent evidence could open up new scenarios for the use of cyanidin derivatives in therapy. Indeed, *Prunus* species are recognized as a highly rich source of pigments and other natural antioxidants, which could be used as dietary supplements beneficial to human health. Future genetic selection programs in order to obtain new *Prunus* varieties are also of interest [18,19].

On the other hand, tart cherries’ health potential, anthocyanins, flavonols and phenolic acids content, as well as antioxidant and anti-inflammatory potential, cardiovascular benefits, the fate of polyphenols in the digestive tract and their impact on gut microbiota, were recently reviewed by Alba et al. [20]. 

As only a limited number of reports exist about sour cherries’ chemical composition [8,9,10,11,12,13], the aim of the present paper is to deepen the knowledge of the polyphenolic profile as tool to support their consumption as healthy food and to valorize and consolidate the tradition of cropping sour cherry tree in the Lazio region for the production of fruits. The production of fruits is dedicated to obtaining processed commercial products including the jam, which is a key ingredient in the preparation of the traditional sour cherry pie (the local name is “crostata visciole di Sezze”), officially recognized within the Italian traditional agrifood products [21].

The experimental design applied was to highlight the effects of technological procedures on the qualitative and quantitative composition of bioactive secondary metabolites. The effects were evaluated using fresh or thawed fruits subjected to two homogenization techniques, before or after the pasteurization. The color analysis performed on the only homogenized samples, as well as HPLC-DAD, the Direct Infusion Electrospray mass spectrometry (DI-ESI-MS) analysis of some selected samples and the anti-radical activity 2,2-diphenyl-1-picrylhydrazyl, DPPH assay performed on the obtained hydroalcoholic extracts, allowed us to determine the optimal processing, as well as to obtain correlation data among the color shown, the phytocomplex profile and the expressed anti-radical activity.

## 2. Materials and Methods

### 2.1. Materials

Plant material, consisting in fully ripe sour cherries fruits (*Prunus cerasus* L. var. *visciola*) freshly harvested in late June, were purchased from “Bontà Natura” (Sezze, Latina, 41°30′ N; 13°04′ E), an Italian farm that directly grows and transforms the fruits. Each bulk contains fruits at different maturation stages. Fruits for experimental procedures were manually selected in order to obtain a homogenous sampling corresponding to maturation stage 4, according to Aslantas et al. [8].

The sampling of the fresh fruits was performed in three consecutive years during the harvesting (June 2019, 2020 and 2021) from the same plantation consisting in plants of 9–11 years.

Part of the sample was immediately processed as fresh fruits and the other part was frozen at −80 °C, stored at −18 °C and defrosted just before the processing. Bi-distilled water, ethanol, methanol, 85% formic acid, 2,2-diphenyl-1-picrylhydrazyl and acetonitrile RS for HPLC were purchased from Merck Life Science (Milan, Italy). 

### 2.2. Sample Processing

**Scheme 1:**
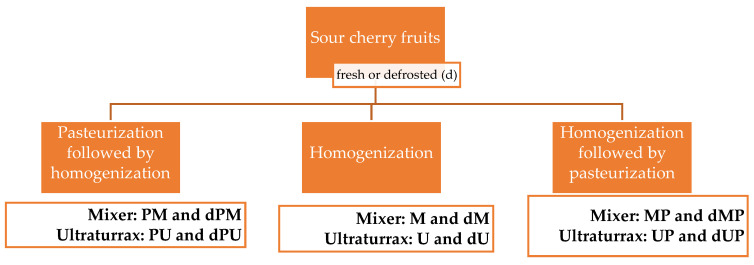
Applied procedures and sample: acronyms. **d**: defrosted samples; **P**: pasteurization; **M** and **U** homogenization by mixer or Ultraturrax, respectively. The acronyms describe the chronological sequence of the applied procedure.

Immediately after the collection, from the commercial bulk the fresh fruits were carefully selected to eliminate the unhealthy ones, and, with a gentle cleaning, residual of stems, peduncles and other impurities were removed. The selected samples (approximately 5 kg for each year collection) were carefully washed and dried on paper. Part of each sample was immediately processed as fresh fruit and the other part was frozen at −80 °C, stored at −18 °C, in the dark, until analyzed, within 6 months. Cold stored samples were defrosted (in the acronym they are identified by the letter **d**) just before the processing. Fresh fruits and the corresponding thawed samples were subjected to four different treatments (Scheme 1). The process starts with homogenization of fruits for 2 min using a domestic mixer (M and dM samples) (Girmi, Modena, Italy) or Ultraturrax^®^ industrial homogenizer (U and dU samples) (VWR, Staufen, Germany). A pasteurization step was applied before (PM, dPM, PU and dPU samples) or after the homogenization process (MP, dMP, UP and dUP samples) by heating at 85 °C for 3 min [22]. On the whole, twelve different treated samples were obtained for each vintage and each experiment was performed at least in triplicate. 

### 2.3. Colorimetric CIEL*a*b Analysis

All the obtained homogenized samples were controlled for their pH value, always ranging between 3.2 and 3.6, and underwent colorimetric analysis. The analyses were conducted using an X-Rite colorimeter SP-62 (Regensdorf, Switzerland), equipped as previously described [23]. Each determination is the mean value from six measurements, performed on both the surfaces of a quartz cell containing the sample homogenates. The results are expressed as the mean value ± standard deviation (SD). CIEL*a*b parameters were calculated as previously reported [24].

### 2.4. Hydroalcoholic Extraction

The homogenized samples were submitted to hydroalcoholic extraction (1:3 *p*/*v*) with a mixture of ethanol/acidified water at 5% acetic acid (70:30 *v*/*v*) for 1 h at room temperature according to Garzoli et al. [4]. Then, the separated solution was concentrated under reduced pressure at 40 °C with a rotary evaporator, freeze dried and stored at 4 °C until further analyses were performed.

### 2.5. Solid Phase Extraction (SPE)

According to Yilmaz et al. [24], with some modifications, the freeze dried hydroalcoholic extracts were submitted to solid phase extraction with a Discovery^®^ DSC-18 SPE Tube column (Merck Life Science, S.r.l., Milan, Italy). After a preconditioning with 5 mL of ethyl acetate, 5 mL of methanol (5% acetic acid) and 5 mL of water, about 200 mg of the extract were dissolved in 3 mL of water (5% acetic acid) and loaded into the column. The column was conditioned with 10 mL of acidified water, washed with 10 mL of ethyl acetate, and finally the anthocyanin fraction was collected with 5 mL of acidified methanol. The obtained methanol fractions were dried under reduced pressure at 40 °C with a rotary evaporator, weighed and stored at 4 °C until further analyses were performed. 

### 2.6. HPLC-DAD Analysis

The hydroalcoholic extracts were solubilized in acidified water (5% acetic acid) at a final concentration of 5 mg/mL and then filtered with a Millex^®^—LG filter (Low Protein Binding Hydrophilic PTFE 0.20 µM Membrane) (Merck Science Life, S.r.l, Milan, Italy). Then, 10 µL of these samples were injected into an HPLC-DAD system made by a Perkin–Elmer apparatus, as previously described [22]. In brief, a Luna RP18 column (5 µ, 250 × 4.60 mm) and a mobile phase, consisting of acetonitrile (A) and acidified water (5% formic acid) (B) in gradient, from 100% to 85% of B in 20 min and then to 35% of B in 30 min. The chromatograms were recorded at 360 nm for flavonols and at 520 nm for anthocyanins identification. The anthocyanins sum was expressed as mg/g fresh weight of cyanidin-3-rutinoside equivalents (y = 16.582x + 34.529; R^2^ = 0.9989), whereas the flavonols’ sum was expressed as mg/g fresh weight of rutin equivalents (y = 13.598x + 33.166; R^2^ = 0.9994). 

### 2.7. DI-ESI-MS Analysis

Four representative samples harvested in 2021, namely dM, dU, dMP and dUP, have been selected and submitted to the DI-ESI-MS analysis implemented with MS/MS measurements. The high structural resolving power of mass spectrometry supported the HPLC-DAD assignments with regard to the anthocyanin fraction. The extracts, prepared as described in Section 2.5, were diluted in water/methanol (1:1 *v*/*v*) to a final concentration of 2 mg/mL and directly infused at 10 μL min^−1^ in the ESI source of a LTQ XL linear ion trap (Thermo Scientific). The source parameters were set as follows (values used for negative mode in the brackets): source voltage = 4.5 (5.00) kV; capillary voltage = 100 (−40) V; capillary temperature = 300 °C; tube lens voltage 75 (−50) V, sheath gas and aux flow rates, 15 and 10, respectively (arbitrary units). Each spectrum, acquired over the 200–1000 *m*/*z* range, arose from averaging 10 full scans, each one consisting of 5 micro scans. A targeted isolation and fragmentation of the compounds characterized by the *m*/*z* ratio predicted anthocyanins and their derivatives, which allowed this investigation to identify seven species in the positive and one specie in the negative polarity, respectively. The precursor ion isolation width was 1–2 Da and the normalized collision energy was set to the value needed to reduce the intensity of the precursor ion to about 10%. The MS-tandem spectra were compared with those collected in literature [25,26,27].

### 2.8. DPPH Analysis

According to Cairone et al., [28], a 100 µM solution of DPPH in methanol was prepared. An aliquot (2.5 mL) was added to 0.5 mL of methanol and monitored by UV/Vis Lambda 25 Perkin Elmer spectrophotometer (Waltham, MA, USA), at 515 nm, until the read absorbance value was stable. Then, 0.50 mg/mL sample solutions were prepared, diluted with 2.5 mL of the previously described DPPH solution and monitored for 30 min to determine the maximum radical scavenging. A calibration curve was constructed using gallic acid as reference (y = 0.998 e^−378^^.^^5x^) and the exerted anti-radical capacity was expressed as milligrams of gallic acid equivalents/g dry extract.

### 2.9. Statistical Analysis

Each assay was replicated at least three times. Data are expressed as mean and are completed by the relative standard deviations, indicated as maximum value found for each series of reported data. Statistical significance (*p*-value) was determined using the XLStat software (New York, NY, USA).

## 3. Results and Discussion

### 3.1. Processing and Color Analyses of Only Homogenized Samples

Sour cherries were directly provided by the farmers during the harvesting in order to obtain fruit samples at the same ripening status as for the commercial production, approximately at the end of June, in three successive years (2019, 2020 and 2021). Immediately transferred in the lab, about a half of the collected samples were at once frozen at −80 °C and then stored at −18 °C until they were thawed and the processing was performed. The workflow (Scheme 1) was based on few steps aiming to simulate some of the most applied procedural treatments, such as homogenization and pasteurization, which were combined in different type and succession. An aliquot of the fresh or thawed cherries was only homogenized in mixer, with the aim to simulate a simple domestic procedure, or in Ultraturrax^®^ homogenizer, to perform an industrial treatment. A third aliquot was pasteurized at 85 °C for 3′ before the homogenization, or pasteurized in the same heat conditions, after the homogenization. Altogether, twelve samples were obtained from fresh and twelve from defrosted cherries (**d** samples) for each vintage and each experiment was performed, at least, in triplicate. The total obtained homogenized samples were subjected to, for example, the color analysis. In this way, we could obtain information by a limitedly modified matrix, not filtered and neither extracted nor purified, in order to obtain the best information about the pigment content. Simplicity, speed and limited cost of color measure allowed for the analysis of a great number of samples, in a very short time, even by unskilled personnel.

The defrosted samples dPU and dU had the highest luminance value (Table 1, 32.25; 32.33 as medium of the three vintages), and the fresh only homogenized M and U showed the lowest ones (28.53; 28.63); dPU represented the sample with the highest a* (redness) and b* (yellowness) values (19.56; 6.96) and M showed the lowest ones (12.04; 3.63). All a* and b* values always fell in the positive region of tristimulus color with a* about three-fold with respect to b*, which corresponds, on the whole, to tonality included in a narrow range between 17.42 (dPM 2020) and 21.12 (UP 2020). The tonality (h_ab_ = arctan b*/a*) does not give information about the pigment content, which is in turn correlated with the absolute values of a* and b* or, even better, with the chroma (C*ab = (a*^2^ +b*^2^)^½^). 

The highest chroma value is 24.83 (**dU** 2019) and the lowest is 6.38 (U 2020), in a very disperse range, which accounts for great differences in pigment content among samples.

Different colorimetric values for sour cherries are reported in the literature, depending on ripeness, cultivar, geographic region, and state of processing and storage [9,29,30]. Horuz et al. [29], who studied the stability of sour cherries under different drying processes (50–70 °C), report a wide range of values (L* 9–20; a* 4–22; b* 0–9). Damar et al. [9] evaluated sour cherry in five maturation stages separating inner and outer parts. Considering the fifth maturation stage (fully ripe samples), only the C*_ab_ parameter was comparable to our data (L* 19–21; C*_ab_ 11–12; h_ab_ 13–15). Finally, Pedisić et al. [30] report values roughly comparable with ours (L* 19–30; a* 10–27; b* 8–13).

**Figure 1:**
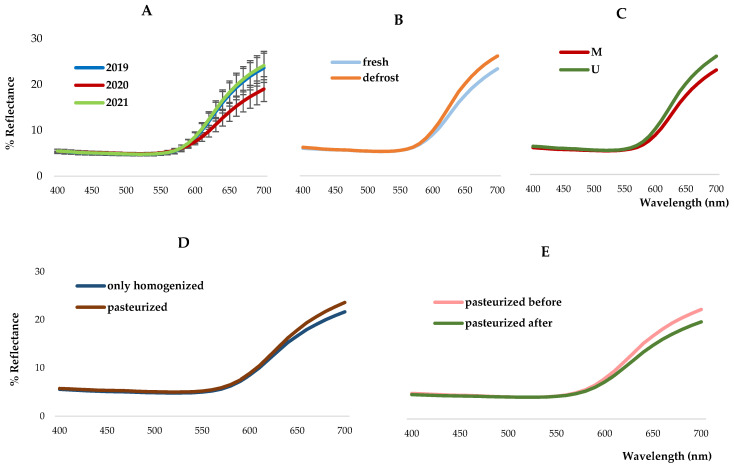
Comparison among reflectance curves of sample clusters. (**A**): mean reflectance curves ± SD of all samples coming from the same harvest year. (**B**–**E**): mean reflectance value of samples belonging to different vintage but subjected to the same procedures.

The 2020 vintage showed a lower pigment content (given as average values, a* is 17.66 in 2019, 12.02 in 2020 and 15.47 in 2021; b* is 6.33 in 2019, 3.90 in 2020 and 5.65 in 2021; C*_ab_ is 18.72 in 2019, 11.77 in 2020 and 16.47 in 2021; *p* value^2019–2020^ < 0.05, *p* value^2020–2021^ < 0.08). In Figure 1 the reflectance curves of the different analyzed samples are reported. The reflectance % represents the color appearance of a sample, expressed in a physical mode, as wavelength absorbed, rather than reflected by a matrix, at wavelength between 400 and 700 nm. Series 2019 and 2021 were richer in anthocyanin pigments with respect to series 2020 (as shown by the lower reflectance curve); series **U** and defrosted samples were richer in pigments, with respect to series M and to not thawed samples. Pasteurization better preserves anthocyanin from degradation if performed before homogenization. As significant differences exist among harvest years, the standard deviations among clusters were generally high and they were not reported. Therefore, the curves should be interpreted as indicative of a general trend shown by the different treated samples. These data will be discussed further through comparison with the HPLC-DAD quali-quantitative results. 

### 3.2. Extraction Procedure and HPLC-DAD Analysis 

**Figure 2:**
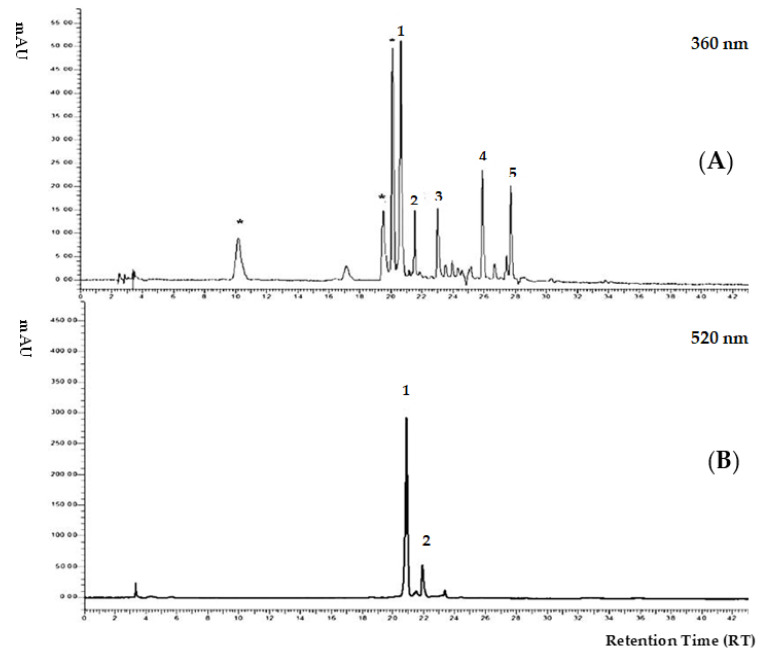
Example chromatograms recorded at 360 nm (**A**) and at 520 nm (**B**). * unknown compounds; 1: CGR; 2: CR; 3: rutin; 4: quercetin-3-galactoside; 5: quercetin.

The HPLC-DAD analysis, performed on the hydroalcoholic extracts, allowed us to identify the flavonoid content of sour cherry samples subjected to hydroalcoholic extraction as previously described [4]. Representative chromatograms related to flavonol and anthocyanin profiles are reported in Figure 2. Flavonols were identified by using reference standards (3, rutin, 4, quercetin-3-galactoside and 5, quercetin) and, when unavailable, by comparison with literature. Concerning anthocyanins, the compound 1 was tentatively assigned to CGR (ref); moreover, compound 2 (CR) was identified by using a reference standard [31,32]. Further support arose from DI-ESI-MS analysis (Section 3.4).

In a study of Kim et al. [33], anthocyanin content was monitored in sweet and sour cherries. CGR as the main component and CR as second component, besides cyanidin-3-sophoroside, cyanidin-3-glucoside and cyanidin-3-rutinoside, were found in sour cherry. Finally, flavonols and flavanols such as catechin, epicatechin, quercetin-3-glucoside, quercetin-3-rutinoside and kaempferol-3-rutinoside were also reported for sour cherries.

Unknown compounds at RT 10.2, 18.6 and 20.4 could be interpreted as procyanidins dimers or trimers, as reported by Woidylo et al. [34]. Procyanidins were not confirmed by our ESI-MS experiments and were not quantified.

Table 2 shows the quantitative data for fresh and thawed samples and related *p*-values. Statistically relevant differences are revealed in the anthocyanins content between **2019** and **2020** vintages, and similar values are shown in 2019 and 2021, both representing the most productive years (anthocyanins sum of 0.42–1.21 mg/g fresh weight in 2019 and 2021 with respect to 0.24–0.59 mg/g fresh weight in 2020). Cya-3-glucosyl rutinoside always soars as the most abundant molecule of all the represented phytocomponents in the analyzed extracts, in agreement with data reported in the literature [9,35].

However, significant differences in terms of absolute content of CGR could be shown, among both different vintages and different treated samples. In particular, it is much more expressed (about 80% of the total anthocyanins) in 2019 and in 2021 vintages, whereas it represents 60% of the total anthocyanin content in 2020 harvest (*p* value, total anthocyanin content 2019 vs. 2020 < 0.05; 2021 vs. 2020 < 0.05).

The flavonols content ranges from a minimum of 0.04 mg/g fresh weight (M and dM 2020) to 0.2 mg/g fresh weight (dMP 2021). Statistically significant differences are again observed comparing 2019 or 2021 with 2020 harvest, which is confirmed as much less productive. 

**Figure 3:**
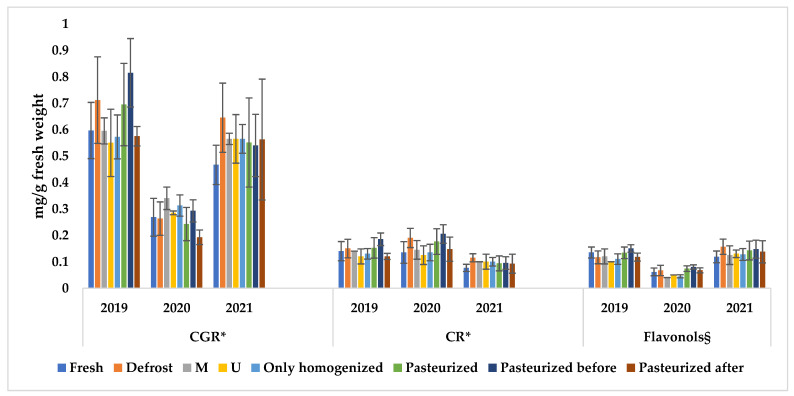
Average anthocyanins and flavonols content in the three vintages and in the different clusters. * Expressed as cyanidin-3-rutinoside equivalents; ^§^ expressed as rutin equivalents.

In Figure 3 the relative contribution of anthocyanin and flavonols to the total polyphenolic content of the three different vintages and in the different clusters is shown. Anthocyanins represent the main component of polyphenols phytocomplex, (CGR alone being 65–70% of the total polyphenols content, except in the 2020 vintage), and flavonols only afford 12–16%, which on the whole is comparable to the CR contents.

HPLC data confirm a higher quality of samples which were submitted to immediate freezing and to pasteurization, especially if performed before the homogenization step, as already shown by the color analysis. The efficacy of pasteurization on the polyphenols oxidase inactivation results in a preservation of the anthocyanin component and the associated pigmentation, as previously reported [4].

Recorded data substantially agree with data found in the literature, where an average anthocyanin content between 0.30 and 1.2 mg/g fresh weight [31,32] and a flavonol content of 0.02–0.07 mg/g fresh weight [26] have been reported.

### 3.3. DI-ESI-MS and MS/MS Analyses

Table 3 reports the polyphenolic and anthocyanin compounds identified and the relevant peak areas.

The most intense peak in all the recorded DI-ESI-MS full spectra invariably corresponds to *m*/*z* 757.33 u [36,37,38]. Its MS/MS spectrum (Figure S1), together with the MS/MS spectrum of *m/z* 595.25, is in full agreement with the spectra of CGR and CR reported in the literature [25,26]. CGR and CR always represent the most abundant molecules detected in all samples, confirming the HPLC-DAD data. 

Besides confirming that the anthocyanin distribution is slightly affected by the specific applied procedure, the DI-ESI-MS measurements allowed us to identify the other represented anthocyanins, which were assigned to cyanidin-3-glucoside, peonidin-3-rutinoside, cyanidin-3-xylosyl rutinoside and pelargonidin-3-glucosyl rutinoside, only visible as traces in HPLC-DAD analysis. The presence of rutin, as a main component of flavonols, was also confirmed. Coumaroylquinic acid, the only compound detected in negative mode, could be tentatively assigned to one of the unknown components shown by the HPLC analysis.

### 3.4. Anti-Radical Activity

Data coming from anti-radical DPPH assays perfectly agree with the previously shown HPLC-DAD data. A high anti-radical potential (Table 4) is shown by the 2019 vintage and similar values are expressed by the 2021 one. A very low activity is exerted by the 2020 vintage, which has a very poor polyphenolic content (average DPPH values 6.5 in 2020, 13.5 in 2021 and 14.8 in 2019). The lowest anti-radical activity is shown by samples M, while the highest one is exerted by the samples dPU.

**Figure 4:**
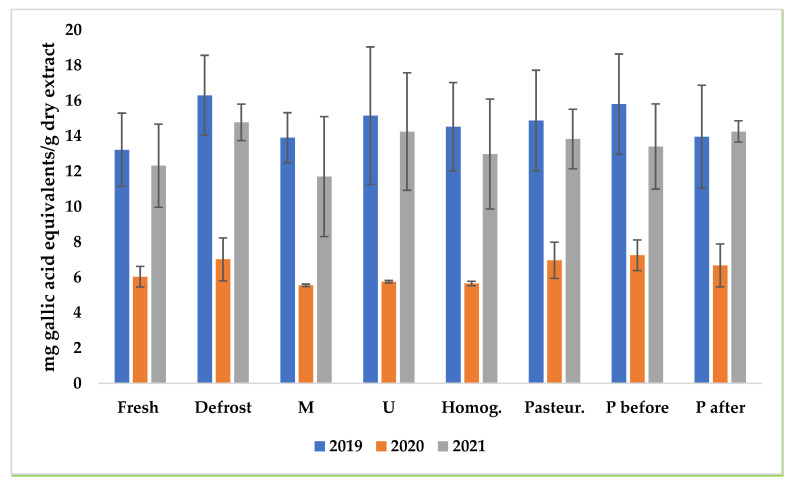
DPPH average values in the three vintages and in the different clusters.

Moreover, in reporting the average values of DPPH in relation to the clusters (Figure 4), all the previously underlined trends were confirmed.

Some selected samples of 2019 harvest (M, dM, U, dU, MP, UP, PM and PU), were also analyzed in terms of ABTS, CUPRAC and FRAP assays. In addition, their activity towards acetylcholinesterase, butyrylcholinesterase, α-glycosidase and α-amylase was assayed (see Materials and Methods and Table S1 in Supplementary Material). The trends in the antioxidant tests confirmed the results obtained by the DPPH assay, but the extracts showed low inhibition abilities on the tested enzymes. As the next vintages, 2020 and 2021 contained a decreased content of polyphenols and a decreased anti-radical activity; they were not assayed with these other anti-radical and enzymatic tests.

On the basis of the collected data, an attempt was made to directly correlate the expressed color, not only to the anthocyanins, but directly to the exerted anti-radical activity. In fact, we aimed to verify if a simple, quick and inexpensive analysis, such color analysis, is able to predict the biomolecule content and the associated health potential. 

As anthocyanins are largely the main represented colored molecules in sour cherries and contribute as absolutely red pigments, a correlation between the colorimeter parameter a* (redness) and the anthocyanin sum evaluated in the different clusters was attempted. Moreover, the chroma value, C*_ab_, a measure of color intensity, was directly interfaced with the DPPH value, representing the health potential, evaluated as anti-radical activity expressed by the hydroalcoholic extracts of sour cherries, differently treated.

**Figure 5:**
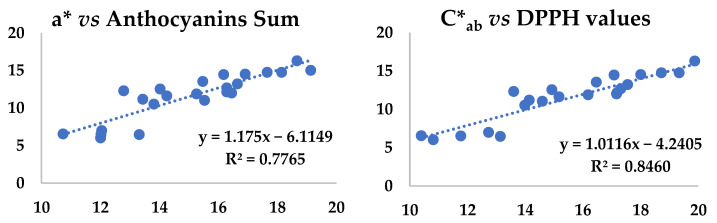
Correlation between a* and anthocyanin sum and between C*_ab_ and DPPH values of different clusters. All values are reported as an average of the selected clusters (vintages 2019, 2020 and 2021; fresh and defrosted 2019, 2020 and 2021; M and U 2019, 2020 and 2021; fresh samples; defrosted samples; only homogenized samples; pasteurized samples; samples pasteurized before or after homogenization process).

As shown by the linear regression graphics (Figure 5), a high correlation exists among a* values and anthocyanin content, revealed by the HPLC analysis. Even a better correlation is shown among the expressed chroma and the exerted anti-radical activity. Color analysis is performed on the homogenates which, not requiring further purified samples, could represent an inexpensive and quick method for a good prevision of the bioactive contents and the related health potential of processed sour cherries.

With the aim to further deepen this aspect, SPE extracts exceptionally enriched in the CGR component (ratio CGR:CR about 12:1 and only traces of other components) were subjected to DPPH analysis in comparison with the available CR standard. CGR exerted an anti-radical activity largely superior (61.37 mg/g gallic acid equivalents) to that expressed by the CR (14.09 mg/g gallic acid equivalents).

## 4. Conclusions

Cropping sour cherry tree for the production of fruits occurs worldwide but somewhere, as in Lazio Region (Italy), where the practice has been consolidated for a long time, the plant is considered part of the local agri-biodiversity and the fruits are ingredients for traditional recipes. Furthermore, the local productive chain is recognized to be of added value due to the marked territorial identity, as confirmed by the local name (visciole di Sezze). In the present research, fruits obtained from local Italian production were investigated and valorized in their composition.

Results showed that a simple processing, based on quick pasteurization, performed before the homogenization step as well as on immediately frozen samples, is able to transform products with minimal impact. The obtained results are of particular concern, both because sour cherries are preferentially consumed as jams or juices and for their peculiar bioactive content. 

A simple, cheap, rapid and non-destructive color analysis was directly performed on the homogenates. Requiring neither filtration, extraction nor purification steps, it was able to predict, with good approximation, the bioactive content and the associated anti-radical potential of the transformed matrices.

The anthocyanins content, determined through HPLC-DAD and DI-ESI-MS, was largely predominant in the polyphenolic content which was shown to be preeminently represented by the cyanidin-3-O-glucosyl rutinoside. This high valuable compound, rarely occurring in nature and actually under evaluation for its AMP activated protein kinase inhibition capacity, showed an exceptionally high anti-radical activity.

Its presence in quantities which can reach 1 mg/g fresh weight, even in case of transformed products, suggests the high potential as functional food of this valuable and underutilized food matrix.

## Figures and Tables

**Table 1:** Colorimetric data of the analyzed samples.

Harvest Year		Type of Sample											
		M	dM	U	dU	MP	dMP	UP	dUP	PM	dPM	PU	dPU
2019	L*	30.99	31.10	31.53	34.94	31.09	31.86	33.38	32.94	32.02	30.66	33.34	31.43
2020		26.84	27.72	26.38	30.26	28.84	30.33	29.35	28.86	31.75	27.72	32.60	31.38
2021		27.77	30.17	27.97	31.78	29.19	31.13	28.71	30.55	27.68	31.22	30.63	33.95
2019	a*	14.46	17.07	16.37	23.18	15.70	16.58	17.85	19.89	16.78	16.51	18.72	18.78
2020		11.95	9.33	11.59	14.85	9.86	1015	10.22	10.41	12.79	10.32	15.63	17.19
2021		9.70	15.54	12.73	18.69	13.58	17.31	13.07	16.33	9.74	18.30	17.92	22.71
2019	b*	4.95	6.13	5.76	8.89	5.61	6.27	6.61	7.65	5.70	5.63	6.38	6.39
2020		2.54	3.08	2.19	5.59	3.55	3.48	3.79	3.36	4.37	3.24	5.62	5.97
2021		3.39	5.59	4.64	6.81	4.93	6.53	4.77	5.94	3.44	6.75	6.52	8.51
2019	C*_ab_	15.29	18.14	17.35	24.83	16.68	17.73	19.04	21.31	17.72	17.44	19.77	19.84
2020		12.22	9.83	11.79	15.86	10.52	10.73	10.91	10.94	13.51	10.81	16.53	18.20
2021		10.28	16.51	13.55	19.89	14.45	18.50	13.92	17.37	10.33	19.50	19.06	24.25
2019	h_ab_	18.78	19.72	19.36	20.99	19.57	20.66	20.25	20.99	18.81	18.83	18.78	18.68
2020		20.94	18.22	20.31	20.63	19.83	18.91	21.12	17.91	18.83	17.42	20.09	19.13
2021		19.24	19.76	20.14	20.02	19.90	20.64	20.12	20.00	19.43	20.27	19.09	20.54

RSD < 1%. L* is the lightness; a* is the redness (positive values) or the greenness (negative values); b* is the yellowness (positive value) or the blueness (negative value); C*_ab_ is the chroma or color in-tensity; h_ab_ is the hue or the color tonality.

**Table 2:** Quantitative HPLC-DAD data of the selected samples, expressed as mg/g fresh weight.

Harvest Year		Type of Sample											
		M	dM	U	dU	MP	dMP	UP	dUP	PM	dPM	PU	dPU
2019	CGR *	0.56	0.63	0.46	0.64	0.56	0.63	0.55	0.56	0.73	0.81	0.72	1.00
2020		0.37	0.31	0.29	0.28	0.22	0.21	0.16	0.18	0.29	0.35	0.28	0.25
2021		0.58	0.55	0.50	0.63	0.48	0.52	0.36	0.89	0.42	0.62	0.46	0.66
2019	CR *	0.14	0.14	0.10	0.14	0.13	0.13	0.11	0.11	0.20	0.17	0.16	0.21
2020		0.12	0.17	0.10	0.15	0.16	0.18	0.08	0.17	0.18	0.24	0.17	0.23
2021		0.10	0.10	0.08	0.12	0.07	0.10	0.06	0.14	0.07	0.12	0.08	0.11
2019	Flavonols ^§^	0.14	0.10	0.10	0.10	0.13	0.11	0.13	0.10	0.15	0.13	0.16	0.16
2020		0.04	0.04	0.05	0.05	0.06	0.08	0.07	0.06	0.07	0.08	0.08	0.09
2021		0.10	0.15	0.12	0.14	0.11	0.20	0.12	0.12	0.10	0.15	0.16	0.18

RSD < 5%; * expressed as CR equivalents; ^§^ expressed as rutin equivalents.

**Table 3:** Anthocyanins and polyphenolic compounds identified by DI-ESI-MS analysis of four selected samples.

	Mass (*m*/*z*)	Peak Area %			
Compound	[M + H]^+^	dM	dMP	dU	dUP
Cyanidin-3-*O*-glu	449.3	3	4	2	3
CR	595.3	12	12	11	12
Peonidin-3-*O*-rut	609.3	7	7	5	4
Rutin	611.2	5	5	4	5
Cyanidin-3-*O*-(2^G^-xyl)-rut	727.2	3	4	2	3
Pelargonidin-3-*O*-(2^G^-glu)-rut	741.2	3	3	2	2
CGR	757.2	66	64	74	72
	**[M − H]^−^**	**dM**	**dMP**	**dU**	**dUP**
Coumaroylquinic acid isomer ^a^	337.2	+	+	+	+

^a^ compound detected in all the analyzed samples in similar amount.

**Table 4:** Anti-radical activity of the analyzed samples.

Harvest Year	Type of Sample											
	M	dM	U	dU	MP	dMP	UP	dUP	PM	dPM	PU	dPU
2019	12.9	14.9	12.4	17.9	15.0	15.4	9.6	15.8	14.9	13.8	14.5	20.0
2020	5.6	5.5	5.8	5.7	5.8	8.2	5.6	7.1	7.1	7.2	6.3	8.4
2021	9.3	14.1	11.9	16.6	13.7	14.0	15.1	14.2	9.9	14.3	14.0	15.4

RSD < 1%.

## Data Availability

Not applicable.

## Supplementary Materials

The following supporting information can be downloaded at: https://www.mdpi.com/article/10.3390/antiox12020445/s1, Materials and methods: Antioxidant and enzyme inhibition assays; Table S1. Biological activity evaluation of selected samples (2019). Figure S1: MS/MS spectrum of the *m*/*z* 757.17 (CGR) and MS/MS spectrum of the *m*/*z* 595.25, (CR). References [39,40] are cited in the supplementary materials.
