# Supplementary material for: Effects of Processing on Chemical Composition of Extracts from Sour Cherry Fruits, a Neglected Functional Food
Source: Antioxidants (Basel). 2023 Feb 10;12(2):445. doi: 10.3390/antiox12020445 (PMC9952311; doi:10.3390/antiox12020445)
Supplement: Supplementary file 1 [file antioxidants-12-00445-s001.zip › antioxidants-2166904-supplementary.pdf]

SUPPLEMENTARY MATERIALS

Effects of processing on chemical composition of extracts from sour cherry fruits, a neglected functional food

Francesco Cairone [1], Caterina Fraschetti [1,*], Luigi Menghini [2], Gokhan Zengin [3], Antonello Filippi [1], Maria Antonietta Casadei [1] and Stefania Cesa [1,*]

[1] Department of Drug Chemistry and Technology, "Sapienza" University of Rome, P.le Aldo Moro 5, 00185 Rome, Italy

[2] Department of Pharmacy, G. d'Annunzio, University of Chieti-Pescara, 66013 Chieti, Italy

3 Department of Biology, Science Faculty, Selcuk University, Konya 42130, Turkey

* * Correspondence: caterina.fraschetti@uniroma1.it (C.F.); stefania.cesa@uniroma1.it (S.C.)

Materials and methods

Antioxidant and enzyme inhibition assays

2,2′-azino-bis(3-ethylbenzothiazoline-6-sulfonic acid) (ABTS) and reducing power (CUPRAC and FRAP) assays. Antioxidant results were expressed as trolox equivalents (TE). The enzyme inhibition towards acetylcholinesterase (AChE) and butyrylcholinesterase (BChE) was determined. The enzyme inhibitory activities of the extracts were calculated as equivalents of the corresponding standard drug per gram of the sample (i.e., galantamine for acetylcholinesterase (AChE) and butyrylcholinesterase (BChE) and acarbose for α-amylase and α-glucosidase inhibition assays).

The experimental procedures for all these assays were comprehensively described in our previous studies [1,2].

Table S1. Biological activity evaluation of selected samples (2019). [a]expressed as mg Trolox equivalents/g dry extract; [b]expressed as mg galamantine equivalents/g dry extract; [c]expressed as mmol ACAE/g dry extract. na: not active

	Selected samples							
	**M**	**dM**	**U**	**dU**	**MP**	**UP**	**PM**	**PU**
**ABTS[a]**	24,8 ± 0.3	47.6 ± 0.4	19.3 ± 0.6	50,1 ± 0.5	22,6 ± 0.4	20,2 ± 0.7	18,3 ± 0.8	17,2 ± 1.1
**CUPRAC[a]**	74,9 ± 0,5	87,2 ± 0,9	60,1 ± 0,2	95,6 ± 1,0	79,5 ± 0,6	72,3 ± 0,3	67,2 ± 0,3	64,9 ± 0,3
**FRAP[a]**	43,8 ± 0,4	50,8 ± 0,9	34,9 ± 0,7	55,9 ± 0,2	47,4 ± 0,2	41,4 ± 0,5	38,8 ± 0,6	39,6 ± 0,2
**AChE[b]**	2.7 ± 0.1	na	2.7 ± 0.1	2,7 ± 0.1	2,7 ± 0.1	2,7 ± 0.1	2,6 ± 0.1	2,5 ± 0.1
**BChE[b]**	2,9 ± 0.1	3,5 ± 0,2	3,1 ± 0,2	3,7 ± 0,3	2,8 ± 0,2	2,6 ± 0,7	3,2 ± 0,1	3,2 ± 0,2
**α-amylase[c]**	0,08 ± 0,01	0,07 ± 0,01	0,07 ± 0,01	0,13 ± 0,05	0,08 ± 0,01	0,09 ± 0,01	0,08 ± 0,01	0,07 ± 0,01

***α*-glucosidase**[c]	1,92 ± 0,01	na	1,90 ± 0.02	na	1,87 ± 0,03	1,95 ± 0,01	1,93 ± 0,01	1,30 ± 0,04

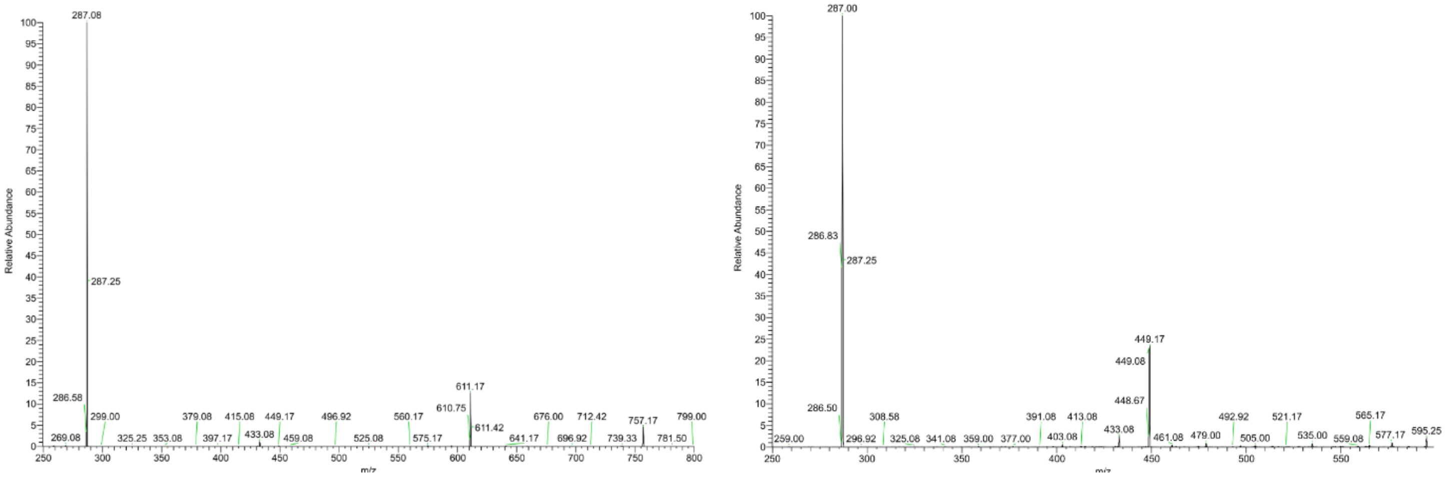

Figure S1. MS/MS spectrum of the *m/z* 757.17 (CGR) and MS/MS spectrum of the *m/z* 595.25, (CR).

References

1. Zengin, G.; Locatelli, M.; Stefanucci, A.; Macedonio, G.; Novellino, E.; Mirzaie, S.; Dvorácskó, S.; Carradori, S.; Brunetti, L.; Orlando, G.; et al. Chemical characterization, antioxidant properties, anti-inflammatory activity, and enzyme inhibition of Ipomoea batatas L. leaf extracts. *Int. J. Food Prop.* **2017**, *20*, 1907–1919.]
2. Chiavaroli, A.; Recinella, L.; Ferrante, C.; Locatelli, M.; Macchione, N.; Zengin, G.; Leporini, L.; Leone, S.; Martinotti, S.; Brunetti, L.; et al. Crocus sativus, Serenoa repens and Pinus massoniana extracts modulate inflammatory response in isolated rat prostate challenged with LPS. *J. Boil. Regul. Homeost. Agents* **2017**, *31*, 531–541.
